# Keystone communities can rescue aquatic metacommunities influenced by pesticide contamination

**DOI:** 10.1002/eap.70145

**Published:** 2025-12-08

**Authors:** Camila Batista Vieira, Gedimar Pereira Barbosa, Ana Carolina dos Santos, Neliton Lara, Erick Mateus‐Barros, Jorge Laço Portinho, Hugo Sarmento, Gilmar Perbiche‐Neves, Cassiana C. Montagner, Luis Schiesari, Victor S. Saito, Tadeu Siqueira

**Affiliations:** ^1^ Departamento de Biodiversidade Instituto de Biociências, Universidade Estadual Paulista (UNESP) Rio Claro São Paulo Brazil; ^2^ Instituto de Desenvolvimento Sustentável Mamirauá Tefé Amazonas Brazil; ^3^ Programa de Pós‐Graduação em Ciências Ambientais Universidade Federal de São Carlos São Carlos São Paulo Brazil; ^4^ Programa de Pós‐Graduação em Ecologia e Recursos Naturais Universidade Federal de São Carlos São Carlos São Paulo Brazil; ^5^ Departamento de Hidrobiologia Universidade Federal de São Carlos São Carlos São Paulo Brazil; ^6^ Instituto de Química, Universidade Estadual de Campinas (UNICAMP) Campinas São Paulo Brazil; ^7^ Escola de Artes, Ciências e Humanidades, Universidade de São Paulo São Paulo São Paulo Brazil; ^8^ Departamento de Ciências Ambientais Universidade Federal de São Carlos São Carlos São Paulo Brazil; ^9^ Centro de Pesquisa em Biodiversidade e Mudanças do Clima, Instituto de Biociências, Universidade Estadual Paulista (UNESP) Rio Claro São Paulo Brazil; ^10^ School of Biological Sciences, University of Canterbury Christchurch New Zealand

**Keywords:** agrochemicals, dispersal, fipronil, mesocosms, metaecosystems, zooplankton

## Abstract

Pesticide contamination in freshwater habitats is a major global issue, affecting water quality, biodiversity, and ecosystem services. Uncontaminated habitats embedded in agricultural landscapes might act as keystone communities, with the ability to restore diversity and ecological processes in contaminated sites through dispersal. Despite their potential relevance, the role of keystone communities in mitigating agrochemical contamination remains untested. We asked if pristine habitats embedded in agricultural landscapes can act as keystone communities and drive the recovery of contaminated habitats. To answer this question, we conducted a mesocosm experiment to simulate zooplankton metacommunity dynamics under three treatments: uncontaminated, fully contaminated, and partially contaminated metacommunities. We examined communities over time following dispersal and pesticide contamination to analyze their trajectories, diversity, and recovery capacity. Analyses were conducted for all species, as well as for Cladocera and Copepoda separately, at both local (individual communities) and regional scales (three communities linked by dispersal—i.e., metacommunities). Taxon‐specific population trajectories indicated that cladoceran densities increased across treatments irrespective of contamination, whereas copepods exhibited species‐level declines or increases under local pesticide exposure. These taxon‐specific population responses to contamination altered community trajectories, resulting in a greater loss of species in completely contaminated metacommunities. Metacommunities with uncontaminated habitats partially recovered from contamination and showed compositional and gamma diversity patterns comparable to uncontaminated metacommunities. Recovery patterns differed across Cladocera and Copepoda, with recovery being more evident at the regional scales. Keystone communities had a greater influence on the recovery of Cladocera community composition and on Copepoda gamma diversity. Our results supported the prediction that keystone communities play a fundamental role in local and regional dynamics of aquatic metacommunities inserted in landscapes with a heterogeneous structure of contamination. Positioning preserved habitats well connected to impacted sites could allow a quick colonization after pesticide contamination, recovering the system until the next crop management cycle. However, taxon‐specific trajectories underscore the need to consider functional and dispersal traits when designing mitigation strategies. We thus suggest a metacommunity perspective for better predictions of risks associated with pesticide use in nature and ways of mitigating them.

## INTRODUCTION

A fundamental characteristic of biological communities is that they are continuously reorganizing in face of natural and human‐induced perturbations (Rumschlag et al., 2023; Spaak et al., 2023). While we still cannot entirely predict how biological communities will respond to future perturbations, there is now sufficient evidence indicating that they have been and will be severely affected, especially through changes in species composition (Jaureguiberry et al., 2022). Despite its high, disproportionate biodiversity (Balian et al., 2008), freshwater ecosystems are under severe threat from human activities (Reid et al., 2019). One of the major threats to freshwater biodiversity is land use change associated with agricultural expansion (Schiesari & Corrêa, 2016). The interconnected hierarchical structure of freshwater ecosystems allows them to both receive and transmit impacts from events occurring on the surrounding landscape. Consequently, localized events within the catchment can influence different parts of the riverine network (Patrick et al., 2021).

Intensive agricultural practices increase the runoff of agrochemicals such as pesticides into aquatic ecosystems, leading to contamination and degradation of water and habitat quality (Allan, 2004). Research on the impact of pesticides on freshwater biota has historically emphasized ecotoxicological assessments, focusing on organismal responses in isolated, monospecific populations under controlled laboratory conditions (Relyea & Hoverman, 2006; Rohr et al., 2006). However, a growing body of work has expanded to community‐level investigations, including mesocosm experiments and field surveys that demonstrate pesticide effects on multispecies assemblages (e.g., Bell et al., 2019; Bendis & Relyea, 2016; Fugère et al., 2020; Hua & Relyea, 2014). For instance, Relyea and Diecks (2008) revealed that repeated low‐dose applications of malathion triggered trophic cascades in aquatic communities, indirectly causing amphibian mortality through zooplankton declines and phytoplankton blooms. Similarly, Almeida et al. (2023) showed that chlorpyrifos exposure reshaped zooplankton dominance hierarchies by differentially affecting species sensitivities, favoring pesticide‐tolerant taxa like *Daphnia magna* over more vulnerable competitors. Despite these advances, understanding of pesticide impacts at broader organizational levels, such as metacommunities embedded in heterogeneous landscapes with varying spatial configurations, remains limited (Hanson et al., 2007; Hayasaka, 2014; Schiesari et al., 2018).

This knowledge gap about the effects of agrochemicals on freshwater ecosystems across spatial scales underscores the urgent need to expand our understanding of biodiversity responses to environmental change under more realistic scenarios (Schiesari et al., 2023). We suggest that in metacommunities (i.e., sets of local communities interconnected via dispersal), the reorganization of biodiversity following contamination should be influenced by both the spatial structure of the metacommunity and the spatial extent of contamination. For example, the impacts of contamination may vary from being localized within specific localities to affecting entire regions, depending on the differences between local communities and their connectivity (Siqueira et al., 2014). Thus, sufficient dispersal between communities would be essential for the reorganization of local communities and, consequently, of entire metacommunities, bringing back locally extinct but regionally present species (Gianuca et al., 2017; Thompson & Gonzalez, 2017).

In a realistic context, where metacommunities are scattered within agricultural landscapes and are unlikely to uniformly experience agrochemical contamination across all local communities, uncontaminated patches should play a major role in the reorganization of contaminated ones. These disproportionately influential patches have been termed keystone communities (Mouquet et al., 2013; Ruhí et al., 2017). Like a keystone species, a keystone community must therefore be disproportionately important for the stability, functioning, and maintenance of other communities in a metacommunity (Mouquet et al., 2013; Ruhí et al., 2017; Yang et al., 2020), maintaining regional diversity and promoting the recolonization of impacted communities and a possible return to a previous state. Despite their crucial ecological importance, the potential role of keystone communities in mitigating agrochemical contamination remains experimentally untested.

Chemical pesticides with insecticide function often lead to increased mortality among primary consumers, particularly sensitive arthropods such as some zooplanktonic microcrustaceans (Hayasaka, 2014; Miller et al., 2020). In these communities, the effects of pesticides can be markedly different between Cladocera and Copepoda (Suzuki et al., 2024). On one hand, some studies indicate that Cladocera exhibit higher tolerance to pesticides than Copepoda (Brendonck & De Meester, 2003; Hanazato, 2001; Suzuki et al., 2024), suggesting that one group may locally dominate biomass and species richness after contamination due to higher population resistance. On the other hand, copepods might be better at recovering from contamination by metals or pesticides, despite documented vulnerabilities such as elevated egg abortion rates in Calanoida (e.g., Rocha et al., 2024) and adverse responses of Cyclopoida to herbicides and ammonia (Di Marzio et al., 2018). These patterns contrast with the low resilience of large limnetic cladoceran species (Palmer et al., 2012; Yan et al., 2004). An emerging question is therefore how the potential differences in resistance and resilience of Cladocera and Copepoda determine their response to pesticides in a metacommunity context, where local extinctions can be balanced by recolonization from keystone communities.

This study delves into the reorganization of communities and metacommunities in response to pesticide contamination, particularly in the presence of potential keystone communities. To do that, we carried out an outdoor experiment using mesocosm zooplankton communities. We hypothesized that pristine habitats in agricultural landscapes act as keystone communities and drive the temporal reorganization of both local communities and metacommunities impacted by contamination. We compared the effects of pesticide contamination on the spatial and temporal dynamics across uncontaminated, partially contaminated, and fully contaminated metacommunities. Specifically, we tested four predictions. First, more sensitive species would exhibit population declines in contaminated habitats, with (1) steeper declines in fully contaminated metacommunities compared to uncontaminated controls and (2) mitigated declines in contaminated habitats of partially contaminated metacommunities due to dispersal from uncontaminated habitats (P1). Second, these population declines would drive a strong directional change in both local and regional species composition, mirroring patterns observed in long‐term studies of copepod succession in polluted reservoirs (Matsumura‐Tundisi & Tundisi, 2003). This would result in communities and metacommunities with similar temporal trajectories (P2). Third, the unique and directional temporal trajectories of local communities within fully contaminated metacommunities (P2) would arise from population declines in sensitive species, leading to reduced local species richness and evenness (P3). These demographic shifts would drive systematic changes in community composition over time. Fourth, over time, metacommunities with keystone communities would recover from contamination and exhibit compositional and diversity patterns similar to those of uncontaminated metacommunities (P4). Our findings partially support these predictions, offering novel insights into how the spatial structure of metacommunities can mitigate contamination effects on local communities, with a particular emphasis on the role of keystone communities.

## METHODS

### Experimental design and sampling procedures

We carried out the experiment on the campus of the Universidade Federal de São Carlos, Brazil, between August and November 2019. Twenty‐seven polypropylene water tanks were filled with 400 L of filtered water. We spatially arranged the tanks into metacommunities so that a tank represented a local community (*n* = 27) and a set of three tanks represented a metacommunity (*n* = 9; Appendix S1: Figure S1).

Spatial connectivity among tanks within each metacommunity was simulated by actively manipulating dispersal. We removed 4 L of water from each tank (1% of the total volume) within each metacommunity, placing it together in a 12‐L container, which was then gently homogenized, and finally proportionally returned to the tanks (Gianuca et al., 2017). This procedure was repeated for all metacommunities. We covered the tanks with 0.75‐mm mesh nets to prevent dispersal from other potential sources.

### Colonizing the tanks with planktonic species

Samples of phytoplankton were taken from the Lobo‐Broa reservoir (−22.18282, −47.89561) and zooplankton from the Fazzari reservoir (−21.970890, −47.887457), in the São Carlos region. We chose these reservoirs because they have a high density and diversity of phytoplankton and zooplankton. We sampled phytoplankton using 20‐μm mesh nets, making eight vertical trawls starting at a depth of 5 m, and filtering approximately 706 L of water for each tank. We sampled zooplankton with 10 vertical drags, starting at a depth of 5 m, using 68‐μm mesh nets, filtering approximately 883 L of water for each tank.

We added phytoplankton samples into experimental tanks on the same day they were sampled from the reservoir. After inoculating phytoplankton, we added nutrients needed to allow population growth (2.5 mL of agricultural fertilizer N:P:K; nitrogen, phosphorus, and potassium) at a 10:10:10 ratio to each tank.

To ensure that the experimental tanks had similar phytoplankton taxonomic composition before the addition of zooplankton, we homogenized the tanks on three occasions: on the 2nd, 8th, and 13th days after phytoplankton was initially added. The homogenization was done by filtering 30 L from each of the 27 tanks with 20‐μm nets, mixing all those samples together in a single 20‐L container, and redistributing them equally back to the tanks.

Zooplankton were added into water tanks on the same day samples were taken from the reservoir, but 15 days after phytoplankton. We carried out two more homogenization events among tanks after inoculating the zooplankton, before starting the experiment. These procedures aimed at starting the experiment with a similar planktonic community composition in all tanks.

### Fipronil contamination

Pesticide contamination was accomplished by applying the insecticide Regent 800 WG (BASF; active ingredient fipronil, 800 g of active ingredient kg^−1^). Fipronil is a broad‐spectrum insecticide, being one of the most consumed active ingredients registered for use on several crops in Brazil (Moutinho et al., 2020). In addition to the high volume and frequency of use in sugar cane fields and other agricultural crops, another advantage of selecting fipronil as a model compound is the availability of official European Union prospective risk assessment reports. Fipronil is also approved for use in the United States, Australia, and certain Member States of the European Union (PPDB, 2024).

Each of the nine metacommunities received one of three treatments (hereafter collectively referred to as “spatial structure of the contamination”): (1) fipronil in the entire metacommunity (fully contaminated), (2) fipronil in two tanks and one uncontaminated tank (partially contaminated, i.e., with a potential keystone community) and (3) a metacommunity free of contamination (uncontaminated). Each treatment was replicated three times (3 tanks × 3 replicates × 3 treatments = 27 tanks).

We defined that the nominal concentration to be applied to the contaminated tanks would be 2 μg/L of fipronil. This concentration falls within the range of measured and predicted field concentrations (Gan et al., 2012; Schiesari et al., 2023). Importantly, we ran a pilot experiment that confirmed that 2 μg/L of fipronil induces detectable changes in zooplankton abundances (e.g., shifts in dominance, reduced richness) without causing widespread extinctions, a critical balance for studying metacommunity recovery dynamics. More information on the rationale for the use of 2 μg/L in this study can be found in Appendix S1.

### Sampling events

Three days after the last homogenization event, we started the experiment and carried out the first sampling event (T1; first day of the experiment; Appendix S1: Figure S1). Zooplankton samples were taken by filtering 30 L of water in each tank with a 20‐μm mesh. We concentrated the filtered water into 5‐mL samples. This sampling event was used as a baseline for comparisons and analyses of the temporal trajectories of communities under different treatments.

On the second day of the experiment, we carried out a first fipronil contamination event (C1) according to the treatments. We prepared a solution of fipronil at a concentration of 2 mg L^−1^ by diluting the commercial powder product Regent(c) in water with a stirrer, of which 200 mL was diluted in each tank. Posterior analyses following Goulart et al. (2020) confirmed that, on average, the fipronil concentration in each tank was 2 μg/L. Quantification of the target compound was performed by liquid chromatography coupled to tandem mass spectrometry (LC–MS/MS). An Agilent model 1200 chromatograph was used.

One day after the contamination event, we carried out a second sampling event (T2) to capture the acute, short‐term response of the zooplankton communities to fipronil contamination. Four days later, we carried out the first dispersal event (D1) among the three communities within each metacommunity. Four days after that, we carried out a third sampling event (T3). Four days after the third sampling event, we carried out another dispersal event (D2), which was followed by a fourth sampling event (T4) 3 days later. We carried out a second contamination event (C2) 2 days after the fourth sampling, and 3 days later, we carried out the fifth sampling event (T5). Two days after the fifth sampling, we carried out another dispersal event (D3). We then carried out the sixth and final sampling event of zooplankton (T6) 2 days after D3. The manipulative phase of the experiment began 30 days after the inoculation and stabilization of the planktonic communities and lasted 25 days (Appendix S1: Figure S1). This duration allows for a few zooplankton reproductive cycles: approximately 2–3 generations for calanoid and cyclopoid copepods (development to maturity ~8–12 days at 22–28°C; Rietzler et al., 2002; Santos‐Wisniewski & Rocha, 2007) and 3–6 generations for cladocerans (8–10 days to maturity; Bomfim et al., 2022; first reproduction in *Bosmina* spp. in ~3.7 days at 20°C; Biswas et al., 2014).

### Zooplankton identification and quantification

Zooplankton taxa were identified using specialized references (Elmoor‐Loureiro, 1997; Perbiche‐Neves et al., 2015; Sendacz & Kubo, 1982) in a stereomicroscope and microscope. Copepodids (juveniles) and adult copepods and cladocerans were counted using acrylic chambers on the stereomicroscope, and nauplii were counted in Sedgewick–Rafter chambers in the microscope. We took a subsample of 1 mL at a time from each sample, which was diluted in water, to identify and quantify copepods and cladocerans, until we obtained a minimum sample of 50 individuals from each group. At the end of the quantification, we recorded the entire quantified volume, measured the unquantified volume, and combined both volumes in the same container. Based on the quantified and unquantified volumes of the sample, we calculated density as the number of individuals per liter.

## DATA ANALYSIS

### Temporal trajectories

To test P1 that sensitive species would decline more in contaminated habitats, especially in fully contaminated metacommunities, we analyzed how pesticide exposure at local and regional scales affected individual species densities over time (see details on modeling below).

To test P2 that contaminated communities would have a more directional change in composition, we applied a community trajectory analysis (CTA) framework centered on the geometric assessment and juxtaposition of community trajectories (temporal variation in species composition). The CTA framework comprises a formal delineation of a community trajectory within a multivariate space, serving as a structured approximation of the dynamics within and among communities (De Cáceres et al., 2019). CTA allows us to explore the geometric attributes inherent in a community trajectory. As such, we calculated two metrics of CTA for three levels of our data: local communities, metacommunities, and treatments.

First, we calculated trajectory length as the sum of segments uniting a trajectory for a given community. This metric indicates the amount of variation in species composition, regardless of direction. Second, we calculated trajectory directionality as a measure of trajectory consistency. This metric takes into account both the length and the angles of a community trajectory. A directional trajectory would have both longer lengths and a smaller sum of angles, which are calculated using circular statistics. We tested for differences in lengths and directionality between treatments (with sites or metacommunities as replicates) by fitting generalized linear models coupled with posterior pairwise comparisons (see details below). For the treatment level, we only explored patterns visually. Differences in trajectory lengths would be interpreted as higher accumulated compositional variation in time for a given treatment, while differences in trajectory directionality would indicate that a given treatment has a more unidirectional change in species composition in time (e.g., a consistent decrease in abundance for a number of species). Changes in community composition were calculated with the Bray–Curtis dissimilarity index. Temporal trajectory analyses were carried out in R with the package ecotraj (De Cáceres et al., 2019).

### Temporal changes in rank abundance curves

To detail how communities and metacommunities changed over time as a response to the spatial structure of the contamination, we investigated their rank abundance curves (RACs). More specifically, to test P3 that population declines in sensitive species would lead to systematic changes in community composition over time, we analyzed if changes in the shape of RACs were due to changes in species richness, in evenness, or a combination of both (Avolio et al., 2019). To do that, we calculated the changes in richness and evenness of communities and metacommunities, between the first (baseline) and last sampling event of the experiment. Changes in species richness were estimated as

(1) $$∆S=\left(S_{t+1}\;-S_{t}\right)/S_{tot},$$

where *S* is the richness of a replicate, *t* is time, and *S*_tot_ is the total number of unique species in both time periods. Since Δ*S* is expressed as a proportion, it ranges from −1 to 1. Larger values indicate greater changes in species richness. A value of −1 or 1 would occur if one of the replicates being compared had no species.

Change in evenness was estimated as

(2) $$∆E=\left(E_{t+1}-E_{t}\right),$$

where *E* is the evenness of a replicate, and *t* is time. Δ*E* also ranges from −1 to 1, with larger negative values indicating greater decreases in evenness. These analyses were carried out using the codyn package (Hallett et al., 2016) in the R program, separately for all groups together, Cladocera and Copepoda, at the local and regional scales.

### Gamma diversity and compositional recovery

To test P4 that, over time, metacommunities with potential keystone communities would recover from contamination and exhibit diversity and compositional patterns similar to uncontaminated metacommunities, we analyzed how regional (gamma) diversity and composition were affected by the spatial structure of the contamination. We first estimated gamma diversity as the number of species per metacommunity using the Hill series of order *q* = 2, for a coverage level of 99% of the sample, with no CI defined. We did that for all groups together and for Cladocera and Copepoda separately. The *q* parameter controls the sensitivity of the measure to species abundance (Hsieh et al., 2016). We used the parameter *q* = 2, which is equivalent to Simpson's diversity index, to consider the effects of the abundance of dominant species on the change in species diversity in metacommunities. Gamma diversity was estimated using the iNEXT package (Hsieh et al., 2016) in the R program (v4.2.1, R Core Team, 2022).

Second, we compared the species composition at the end of the experiment (T6) among treatments using permutational multivariate ANOVA (PERMANOVA; Anderson, 2001). When the global model was associated with a *p* value <0.05, we conducted pairwise comparisons among all treatments. PERMANOVA is a nonparametric method and was conducted using the vegan package (Oksanen et al., 2007) in the R program (v4.2.1, R Core Team, 2022). Because PERMANOVA was used to complement gamma diversity analyses, it was only applied to the local scale (communities) for simplicity.

### Generalized linear models

We used generalized linear models (GLM) and mixed‐effects models (GLMMs) with a Gaussian distribution to test the relationship between our response (population density; temporal trajectory metrics; metrics of compositional change; gamma diversity) and explanatory variables (spatial structure of contamination; time). For the species‐level GLMM, we included density as the response variable and species identity, time, and their interactions with contamination levels (local contamination and regional contamination) as fixed effects, with random effects accounting for mesocosm and metacommunity identity. For the models in which trajectory and RAC change metrics were the response variables, time was not included as a predictor, as the response variable already represented temporal variation. For gamma diversity, we built the models for each response variable, testing the main effects of time and spatial structure of contamination (both treated as categorical) in an interactive and additive way. When an interaction model was not associated with a *p* value <0.05, we readjusted it to an additive fit.

When we found a relationship between a response and explanatory variables, we ran specific pairwise comparisons (contamination treatments against each other). For the analysis of trajectory metrics, when one of these pairwise differences included the partially contaminated treatment, we ran an additional GLM and pairwise comparison with local contamination treatment nested within metacommunity treatments. This was done to identify differences between contaminated and keystone communities within the partially contaminated treatment only. When an interaction between time and spatial structure of contamination was associated with a *p* value <0.05, we compared contamination treatments with each other, but only at the initial (T1) and final (T6) sampling events. But when there was only a relationship with the spatial structure of contamination, we compared the contamination treatments with each other. When there was a relationship with time only, we did not perform pairwise comparisons. GLM(M)s were fit using the R packages lme4 (Bates et al., 2014) and glmmTMB (Brooks et al., 2017). Pairwise comparisons were made with the package emmeans (Lenth & Lenth, 2018).

## RESULTS

We found 22 microcrustacean taxa, 11 of which were Cladocera and 11 Copepoda. Cladocera were represented mainly by the species *Bosmina freyi*, *Moina minuta*, *Ilyocryptus spinifer*, and *Macrothrix* cf. *squamosa*. Copepods were predominantly represented by Cyclopoida nauplii, Calanoida nauplii, and copepodites.

### Temporal trajectories

**FIGURE 1:**
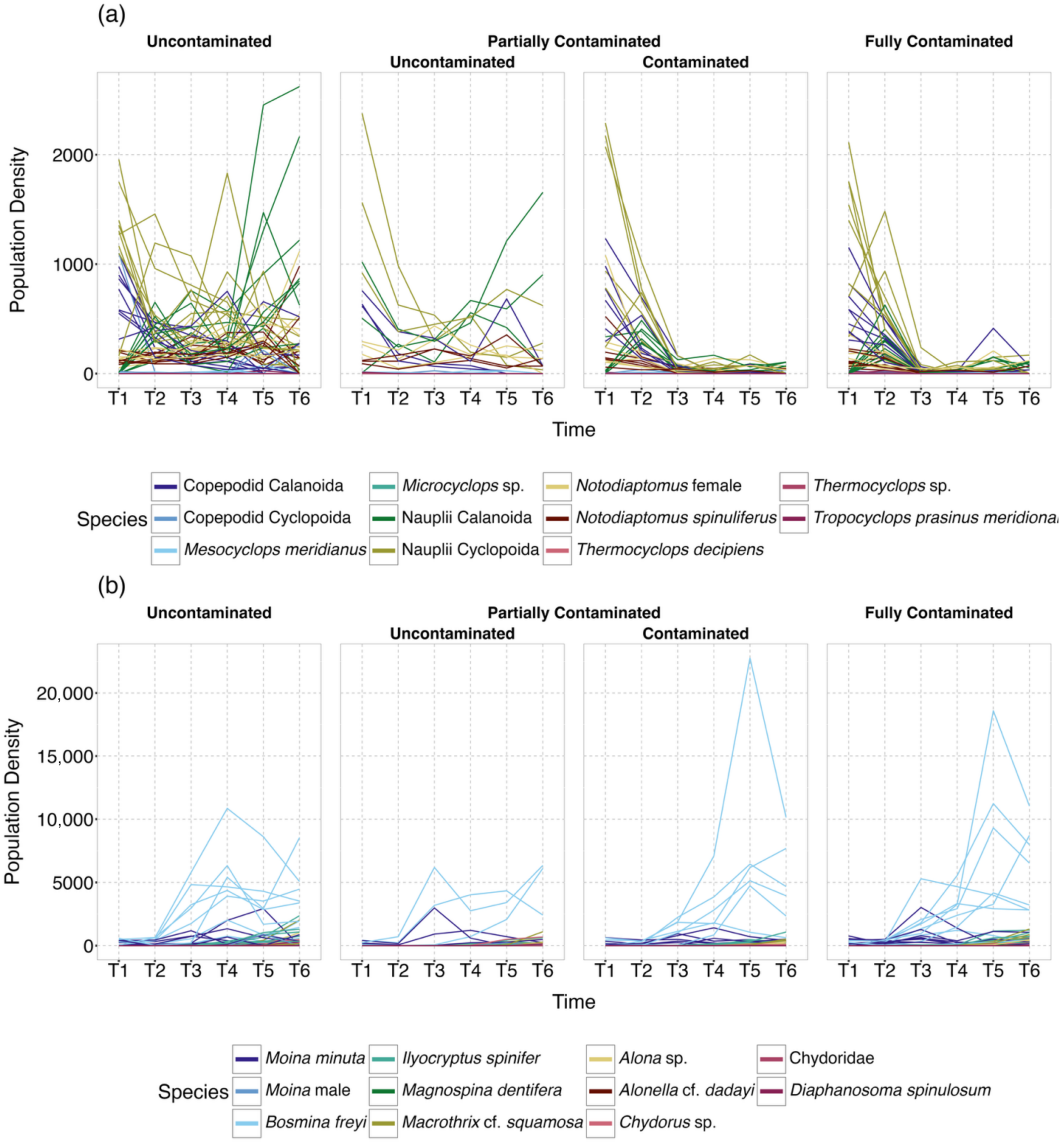
Temporal trajectories of population densities for (a) Copepoda and (b) Cladocera across different contamination treatments in individual mesocosms. Each panel represents a combination of local contamination status (uncontaminated or contaminated) and regional contamination level (uncontaminated, partially contaminated, or fully contaminated). Lines represent individual replicates, and species are color‐coded.

We found that many copepod taxa declined under local contamination while a few were not affected, highlighting context‐dependent sensitivity (Figure 1a). In contrast, cladocerans showed no clear response to contamination, with densities generally increasing over time across treatments (Figure 1b). Indeed, a graphical exploration of aggregated densities of Cladocera and Copepoda indicated that these groups had very different responses to contamination (Appendix S1: Figure S2). To address high variation between taxonomic groups, we initially attempted a combined model for Cladocera and Copepoda while accounting for group and species identity, but it failed to converge due to overparameterization. Subsequent separate models for each group confirmed divergent responses: both groups exhibited species × time interactions while only copepods exhibited a species × local contamination interaction (Table 1). Different from what we expected (P1), we found no evidence that the spatial structure of contamination (uncontaminated, partially contaminated, or fully contaminated metacommunities) influenced species‐level densities (Table 1), indicating that local contamination drives taxon‐specific responses in copepods, but regional connectivity did not buffer populations as hypothesized.

**TABLE 1:** Changes in population densities.

Term	χ^2^	df	*p* value
Copepoda			
Regional contamination (RC)	3.062	2	0.216
Species identity	1426.990	10	<2.2 × 10^−16^
Time	202.613	5	<2.2 × 10^−16^
Local contamination (LC)	32.277	1	1.337 × 10^−8^
Species identity × RC	8.834	20	0.985
Species identity × Time	1282.911	50	<2.2 × 10^−16^
Species identity × LC	85.605	10	3.966 × 10^−14^
Cladocera			
Regional contamination (RC)	0.087	2	0.957
Species identity	1292.345	10	<2 × 10^−16^
Time	113.705	5	<2 × 10^−16^
Local contamination (LC)	0.006	1	0.941
Species identity × RC	4.198	20	0.999
Species identity × Time	733.619	50	<2 × 10^−16^
Species identity × LC	5.559	10	0.851

*Note*: Summary statistics of generalized linear mixed‐effects models (GLMM) with Gaussian distributions for the differences between treatments at local and regional (metacommunity) scales.

Abbreviation: χ^2^, chi‐square statistic.

**FIGURE 2:**
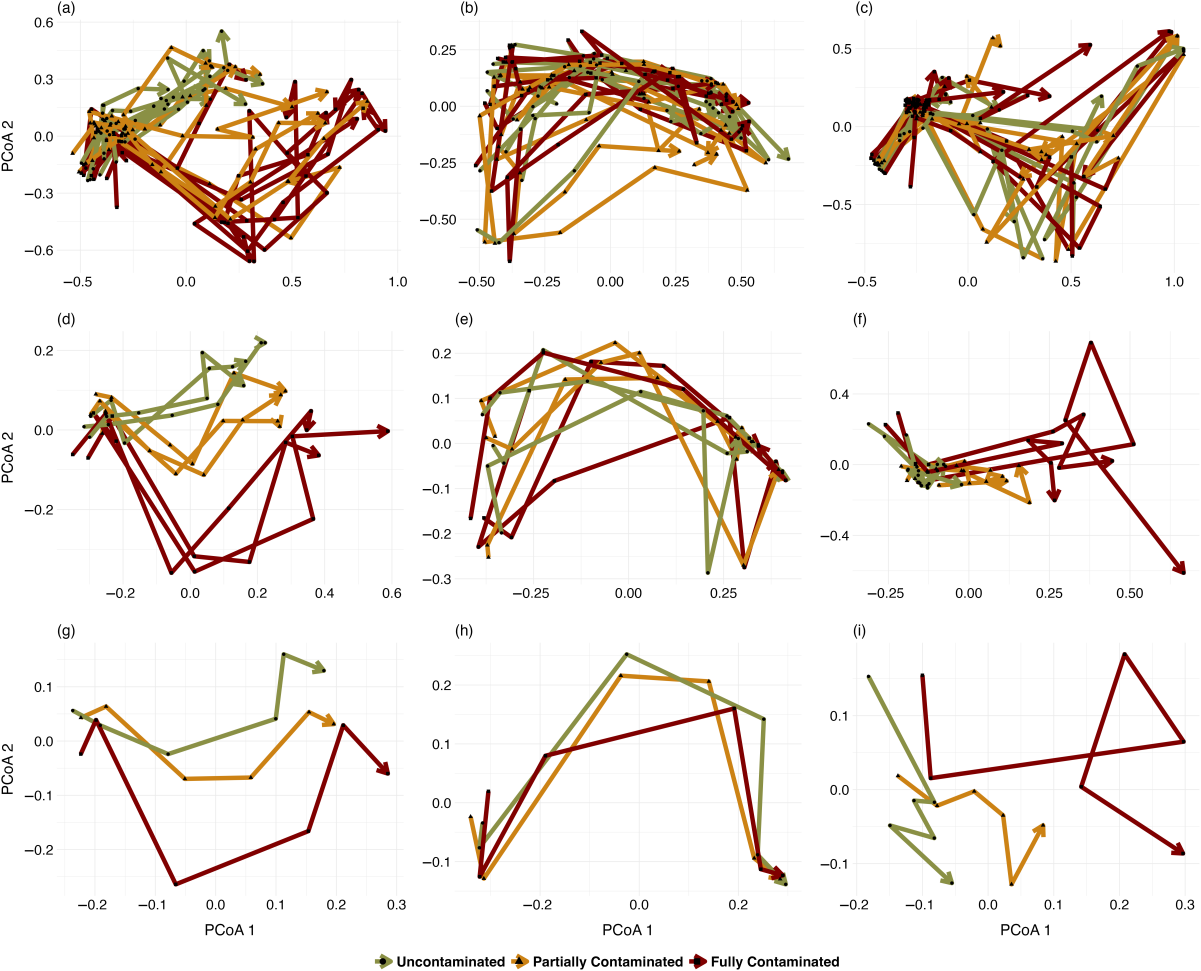
Principal coordinate analysis (PcoA) plots depicting the trajectories of communities (a–c), metacommunities (d–f) and whole treatments (g–i) in experimental mesocosms using Bray–Curtis dissimilarity for the whole set of taxa (a, d, g), Cladocera only (b, e, h), Copepoda (c, f, i). Each treatment was composed of three replicated metacommunities, each one with three communities connected by dispersal. Metacommunities and whole treatment trajectories were presented using the summed abundance in local communities. Arrows represent the direction of community change following six sampling events along the experiment.

Trajectory metrics (length and directionality) depicted similar patterns at the local and regional scales, but different patterns when we analyzed the entire zooplanktonic community or Cladocera and Copepoda separately. For the whole set of taxa at the local scale, the temporal trajectory of communities within uncontaminated metacommunities tended to differ from those within fully contaminated ones (Figure 2a). The temporal trajectory of communities within partially contaminated metacommunities (with potential keystone communities) tended to vary more among each other (Figure 2a,d). The trajectories of communities in uncontaminated habitats within partially contaminated metacommunities (i.e., potential keystone communities) were more similar to communities within uncontaminated metacommunities (Appendix S1: Figure S3).

For the whole set of taxa (Cladocera and Copepoda together), we found that trajectory lengths differed among treatments. Supporting our P2, communities within fully contaminated metacommunities had longer trajectories (accumulated temporal beta diversity) in comparison to those within partially contaminated (Estimate = −0.47, SE = 0.15, df = 24, *t* = −3.00, *p* = 0.016) and uncontaminated metacommunities (Estimate = −0.93, SE = 0.15, df = 24, *t* = −5.86, *p* < 0.001; Figure 2a). However, communities within partially contaminated metacommunities also had longer trajectories than those within uncontaminated metacommunities (Estimate = 0.45, SE = 0.15, df = 24, *t* = 2.85, *p* = 0.022). For that case, we ran a second GLM with local contamination nested within metacommunity contamination. This model indicated that locally contaminated communities had longer trajectories than uncontaminated ones, independently of the metacommunity treatment (Appendix S1: Table S1). This result indicates that our hypothesized keystone communities did not alter the amount of compositional variation in locally contaminated communities, in contrast to our P2.

Also in contrast with P2, trajectory directionality differed among treatments, with less directional trajectories in communities within fully contaminated than in uncontaminated (Estimate = 0.07, SE = 0.02, df = 24, *t* = 2.94, *p* = 0.018) and partially contaminated metacommunities (Estimate = 0.06, SE = 0.02, df = 24, *t* = 2.50, *p* = 0.049). This result indicates that fully contaminated communities had a less consistent pattern of change over time, likely due to the contamination events altering assembly dynamics (Figure 2a). The second GLM for that case indicated differences between fully and uncontaminated treatments. Directionality was not different between contaminated and uncontaminated communities within partially contaminated metacommunities (Appendix S1: Table S2), confirming that uncontaminated communities acted as keystone communities by altering the temporal dynamics of locally contaminated communities (as we expected; P2).

At the metacommunity scale (temporal trajectory of whole metacommunities), we observed the same tendencies as at the local scale, but with a clearer gradual separation in the visual trajectories between treatments (Figure 2d–f). Partially contaminated metacommunities were in between the two other treatments (Figure 2d). Despite these visual tendencies, we did not observe statistical differences among treatments in their trajectory lengths or directionalities, probably due to low statistical power (*n* per treatment = 3).

Trajectory patterns were visually different for Cladocera and Copepoda. Cladocera showed a stronger signature of temporal changes, with much weaker differences among treatments (Figure 2b,e,h). In contrast, Copepoda had trajectories indicating more variation in fully contaminated metacommunities (Figure 2c,f,i). However, we did not observe statistical differences among treatments in the models for either Cladocera or Copepoda.

### Metrics of compositional change

In partial agreement with P3, temporal variation in rank‐abundance curves between the initial and final sampling events was due to changes in species richness only. Temporal changes in evenness were not related to the spatial structure of the contamination for any of the models we ran.

**FIGURE 3:**
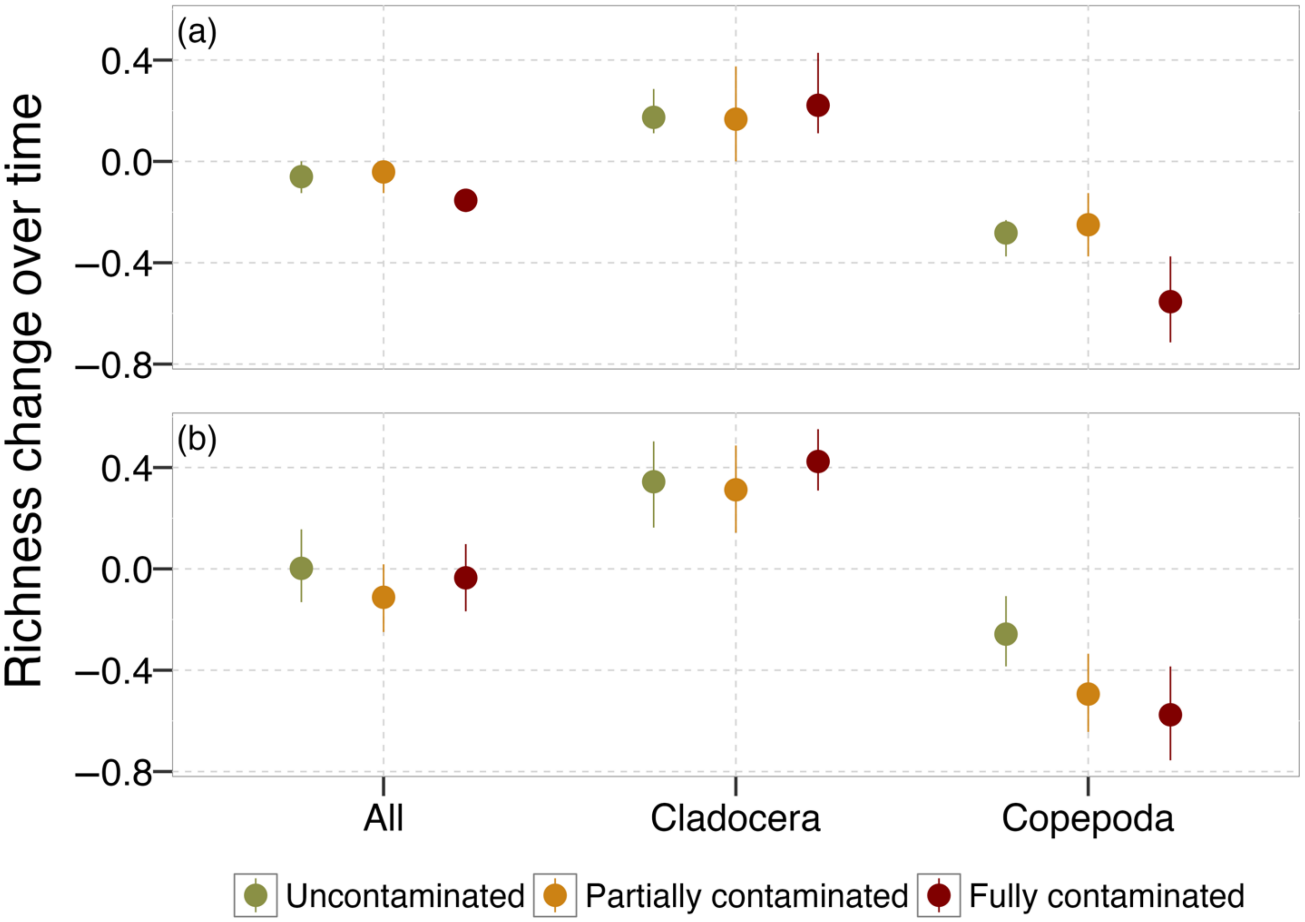
Regional (a) and local (b) changes in richness over time using rank abundance curves for whole communities and separately for Cladocera and Copepoda (regional changes depict the aggregated [summed] abundances of three local communities forming one metacommunity [*n* = 3]). Changes in species richness were estimated following Equation (1). Since Δ*S* is expressed as a proportion, it ranges from −1 to 1.

For Copepoda, the magnitude of the difference in species richness was affected by the spatial structure of the contamination, both locally and regionally (Figure 3, Table 2). Pairwise comparisons indicated that local communities within fully contaminated metacommunities lost more species than those within uncontaminated metacommunities (Appendix S1: Table S3). Different from expected, changes in local species richness did not differ between partially and fully contaminated metacommunities (Figure 3b; Appendix S1: Table S3). As a stronger support for P3, the role of keystone communities manifested at the regional scale (Figure 3a). As expected, changes in regional species richness were similar in uncontaminated and partially contaminated metacommunities, but different in fully contaminated ones (Appendix S1: Table S3). Fully contaminated metacommunities lost more species than those in uncontaminated and partially contaminated metacommunities (Figure 3a). Changes in the species richness of all groups or Cladocera were not influenced by the spatial structure of the contamination at any spatial scale (Figure 3, Table 2).

**TABLE 2:** Changes in species richness.

Group	Scale	Test statistic	Estimate	*p* value	*R* ^2^
All	Local	Chisq	1.18	0.55	0.043
	Regional	LR Chisq	5.93	0.051	0.497
Copepoda	Local	Chisq	7.28	**0.026**	0.219
	Regional	LR Chisq	9.76	**0.0076**	0.619
Cladocera	Local	Chisq	0.87	0.647	0.032
	Regional	LR Chisq	0.21	0.903	0.033

*Note*: Summary statistics of generalized linear models (GLM) with Gaussian distributions for the differences between treatments at local and regional (metacommunity) scales. *p* values were estimated based on 2 df. Bold values indicate statistical differences.

Abbreviations: Chisq, chi‐square; LR Chisq, likelihood ratio chi‐square.

### Gamma diversity and compositional recovery

**FIGURE 4:**
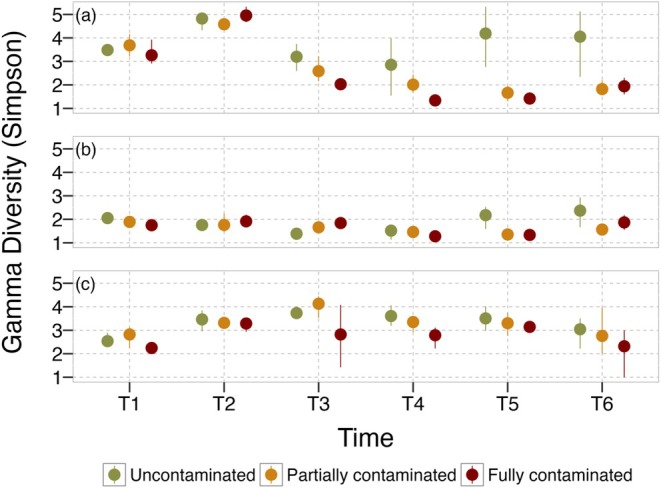
Gamma diversity (Simpson) of all taxa together (a), Cladocera (b), and Copepoda (c) over time in uncontaminated (green), partially contaminated (yellow), and fully contaminated (red) metacommunities. Bars indicate 95% CIs and dots indicate the mean.

We found that variation in the gamma diversity of all groups (Cladocera and Copepoda together) depended on an interaction between the spatial structure of the contamination and time (LR Time × Contamination_10,36_ = 29.26; *p* = 0.0011; *R* ^2^ = 0.82; Table 3). In the last sampling event (T6), the gamma diversity of zooplankton was higher in uncontaminated metacommunities than in those that were partially (with potential keystone communities) and fully contaminated (Figure 4a; Appendix S1: Table S4). Different from what we expected (P4), the gamma diversity of zooplankton was similar in the partially and totally contaminated metacommunities at the end of the experiment (Figure 4a; Appendix S1: Table S4). These results were mainly determined by the response of cladocerans to the spatial structure of the contamination. The global PERMANOVA for the whole community indicated that community composition between treatments differed at the last sampling event (pseudo‐*F* = 5.392, df = 2, *p* = 0.001, *R* ^2^ = 0.31) and among all pairs of treatments (pseudo‐*F* > 2.16, df = 1, *p* < 0.037, *R* ^2^ > 0.11).

**TABLE 3:** Changes in gamma diversity.

	LR	df	*p* value	*R* ^2^
All				
Time	17.7202	5	0.0033	0.823
Contamination	0.6094	2	0.7373	
Contamination × Time	29.2591	10	0.0011	
Cladocera				
Time	28.9064	5	0.0001	0.628
Contamination	1.7841	2	0.4098	
Contamination × Time	29.3411	10	0.0011	
Copepoda				
Time	21.6182	5	0.0006	0.407
Contamination	9.9071	2	0.0071	

*Note*: Summary statistics of generalized linear models (GLM) with Gaussian distributions for the effects of treatments and time.

Abbreviation: LR, likelihood ratio.

The gamma diversity of Cladocera also depended on an interaction between the spatial structure of the contamination and time (LR Time × Contamination_10,36_ = 29.34; *p* = 0.0011; *R* ^2^ = 0.63; Table 3; Figure 4b). In the last sampling event (T6), the gamma diversity of Cladocera was higher in metacommunities without contamination than in those that were partially contaminated, but similar in the partially and totally contaminated metacommunities (Figure 4b; Appendix S1: Table S4). The general PERMANOVA for Cladocera indicated a lack of compositional difference among treatments (pseudo‐*F* = 0.967; df = 2, *p* = 0.539; *R* ^2^ = 0.074).

The gamma diversity of Copepoda was related to the spatial structure of the contamination and to time, but independently (LR Time_5,46_ = 21.62; *p* = 0.0006; LR Contamination_2,46_ = 9.91; *p* = 0.007; *R* ^2^ = 0.41; Figure 4c). As expected (P4), pairwise comparisons indicated that the gamma diversity of Copepoda was similar in the uncontaminated and partially contaminated ones, and lower in the totally contaminated metacommunities (Appendix S1: Table S4). This result indicates that uncontaminated communities acted as keystone communities by maintaining the gamma diversity of Copepoda similar to that of the uncontaminated metacommunities (Figure 4c). In contrast to that, the general PERMANOVA for Copepoda indicated a statistical difference in species composition among treatments (pseudo‐*F* = 4.605, df = 2, *p* = 0.001, *R* ^2^ = 0.27) and also among all pairwise comparisons (pseudo‐*F* > 2.3794, df = 1, *p* < 0.036, *R* ^2^ > 0.12946).

## DISCUSSION

Our experimental study reveals how aquatic metacommunities that differ in their spatial structure reorganize locally and regionally in response to pesticide contamination. We found that population density trajectories diverged between taxonomic groups. Cladocerans showed no directional response to contamination, while copepods exhibited species‐specific declines under local pesticide exposure. Population‐level responses to pesticide contamination modified community temporal trajectories, resulting in communities and metacommunities with less directional temporal trajectories when contaminated by pesticides, thus deviating from the systematic turnover observed for uncontaminated communities. These non‐directional temporal trajectories of communities within fully contaminated metacommunities were mostly due to the loss of species over time, likely as a consequence of contamination events. We confirmed the role of uncontaminated patches as keystone communities. Metacommunities with keystone communities partly recovered from contamination events and exhibited compositional and diversity patterns similar to those of uncontaminated metacommunities, but that differed among Cladocera and Copepoda. The absence of regional contamination effects on population‐level densities suggests that emergent patterns in alpha, beta, and gamma diversity, as well as temporal trajectories (e.g., recovery mediated by dispersal from uncontaminated patches), were not reducible to individual species trends. Together, our results provide novel evidence supporting the expectation that keystone communities can play a fundamental role in the local and regional dynamics of aquatic metacommunities in landscapes with a heterogeneous structure of contamination, while underscoring the importance of taxonomic identity in shaping resilience to anthropogenic stressors.

### Temporal trajectories

Although mesocosm and field studies have long shown that pesticides can trigger community‐level shifts, from zooplankton‐mediated trophic cascades (Relyea & Diecks, 2008) to altered dominance hierarchies favoring tolerant taxa (Almeida et al., 2023), our models show that such patterns emerge via species‐specific responses to local pesticide exposure. Copepods ranged from steep declines to unexpected increases under contamination, while cladocerans exhibited neutral or positive trends across treatments. These divergent trajectories were driven by local responses and remained unaffected by the broader spatial arrangement of contaminated and uncontaminated patches. Still, species‐specific responses capture only part of the picture. Emergent compositional dynamics could not be inferred from species‐level trends alone, underscoring the value of integrating species‐specific and community‐level perspectives.

The presence of keystone communities in partly contaminated metacommunities influenced both regional and local dynamics of zooplankton, sometimes making them indistinguishable from uncontaminated (meta)communities. At the regional scale, keystone communities caused partially contaminated metacommunities to follow similar temporal trajectories in species composition to those of uncontaminated metacommunities. While such similarities in regional dynamics could have emerged as a consequence of a large‐scale averaging effect (Tilman, 1999), we also found that, at the local scale, trajectory directionality within partly contaminated metacommunities was similar for contaminated and keystone communities. We suggest that dispersal from keystone uncontaminated communities mitigated pesticide impacts in the contaminated local communities. These results indicate a potential key role of preserved water bodies inserted in agricultural landscapes by dispersing species and avoiding the general loss of species observed in contaminated metacommunities, a spatial dynamic that can stabilize ecosystem functioning and structure (Schiesari et al., 2023). Keystone communities are thought to have disproportionate effects on the dynamics and structure of metacommunities (Mouquet et al., 2013) and our results indicate that this can be a useful concept for management and conservation strategies. Within a metacommunity approach (Schiesari et al., 2019), positioning a preserved community well connected to impacted sites, would allow a quick colonization after pesticide contamination, at least partially recovering the system until the next agricultural management cycle. One of the main challenges ahead is figuring out how to translate our results to real‐world landscapes. That includes identifying the position of and amount of keystone communities in a way that promotes their connectivity to impacted ones without jeopardizing them to direct impacts.

### Differences between Cladocera and Copepoda

Previous studies emphasized how cladocerans can be more resistant to pesticides while copepods can recover quicker after local extinctions (Palmer et al., 2012; Suzuki et al., 2024; Yan et al., 2004). In our study, the main differences in temporal dynamics among treatments were driven by copepod population declines under local pesticide exposure. While cladocerans consistently changed over time in all treatments, as expected by deterministic community assembly models (De Cáceres et al., 2019), Copepoda varied more in contaminated communities in comparison to uncontaminated ones. These results reinforce the idea of a lower resistance of copepods.

However, although Cladocera was less influenced in general by pesticide contamination, their final gamma diversity was lower in the partially and fully contaminated metacommunities, in contrast to Copepoda, which had the same gamma diversity for partly and uncontaminated treatments and distinct community composition, as a result of dispersal recolonizations from keystone communities. These findings indicate that Copepoda indeed had lower resistance, but also higher resilience to pesticides in terms of their diversity when dispersal is sufficient. Cladocera was mainly composed of the resistant *B. freyi*, *M. minuta*, and *I. spinifer* with some other rare species, which made their composition (particularly the three core species) stable among treatments, while losing the rare species with pesticides. Copepoda in contrast was mainly composed of one species in the adult stage (*Notodiaptomus spinuliferus*) and multiple rarer species in the adult stage; there was also a high abundance of immature individuals. Over time, less abundant adults disappeared across all treatments while the abundance of immature individuals declined with pesticide contamination, leading to a major shift in community composition. Such detailed differences in the diversity and composition of Copepoda and Cladocera emphasize the need to use multiple dimensions of biodiversity to better understand anthropogenic impacts. For example, had we used diversity or composition only, we would have reached opposite conclusions about the impacts of pesticides and the role of keystone communities for these two groups. Species diversity and composition are two connected properties of biological communities and both can be associated with ecosystem functioning (Schiesari et al., 2023; Tilman et al., 2014) and services (Isbell et al., 2011; Pritsch et al., 2023). Given that the Cladocera and Copepoda are two major groups of plankton, their distinct response adds mounting evidence for the complex impact of pesticides in aquatic communities that may have consequences for entire ecosystems.

Our study shows a positive effect of keystone communities on the diversity and structure of contaminated metacommunities. While this can be viewed as a way to recover and mitigate impacts in freshwater ecosystems, there are a few limitations in our study that need to be considered. Our experimental simulation of metacommunity dynamics is a simplification of multiple ways freshwater communities can be connected. For example, in heterogeneous landscapes, freshwater ecosystems can exchange water and organisms in a directional way (upstream to downstream) and not in the multidirectional way we did. Also, dispersal can happen via animal vectors (e.g., birds) and resistance eggs, making colonization more complex and not necessarily density dependent (Havel & Shurin, 2004). We simulated metacommunities with simplified spatial structure—that is, one uncontaminated site connected to two contaminated ones. In real‐world landscapes, anthropogenic impacts are manifested in more complex ways, usually as nonlinear gradients, with pristine ecosystems being rare. Given that we simulated a moderate level of contamination (2 μg/L of Fipronil), it can actually be considered an optimistic scenario in mechanized agriculture regions (Miller et al., 2020); the dynamics happening in real‐world agricultural landscapes could be much worse. Therefore, both the contamination setting and dispersal dynamics can modify the role of keystone communities and deserve attention in future studies.

Our experimental study shows that the presence of pesticides in freshwater ecosystems, even at concentrations accepted by some environmental agencies, changes the local and regional composition of biological communities. Pesticide contamination caused changes in the persistence of some species, which consequently altered the temporal trajectory of communities and ultimately led to changes in the regional pool of metacommunities. However, our results indicate that the spatial structure of contamination matters. One of the implications that emerges from our findings is that uncontaminated communities in regions where contamination is widespread may function as keystone communities, at least partially compensating for the negative effects caused by contamination. We suggest that an explicit consideration of both local and regional dynamics is essential for more robust predictions of the risks associated with pesticide use in nature.

## CONFLICT OF INTEREST STATEMENT

The authors declare no conflicts of interest.

## Supporting information


Appendix S1.

## Data Availability

Data and code (Batista Vieira et al., 2025) are available in Zenodo at https://doi.org/10.5281/zenodo.17202011.
