# Supplementary material for: Keystone communities can rescue aquatic metacommunities influenced by pesticide contamination
Source: Ecol Appl. 2025 Dec 8;35(8):e70145. doi: 10.1002/eap.70145 (PMC12683696; doi:10.1002/eap.70145)
Supplement: Supplementary file 1 — Appendix S1. [file EAP-35-e70145-s001.pdf]

Journal: Ecological Applications

Appendix S1

Keystone communities can rescue aquatic metacommunities influenced by pesticide contamination

Camila Batista Vieira, Gedimar Pereira Barbosa, Ana Carolina dos Santos, Neliton Lara, Erick Mateus Barros, Jorge Portinho, Hugo Sarmento, Gilmar Perbiche Neves, Cassiana C. Montagner, Luis Schiesari, Victor S. Saito, and Tadeu Siqueira

Section S1: Methods

Below, we synthesize evidence from predicted environmental concentrations, field experiments, and environmental monitoring to contextualize our chosen concentration of 2 µg/L of fipronil.

a) Predicted Environmental Concentrations (PECs):

Based on recommended application rates for fipronil (80–400 g/ha, as per Regente 800 WG product guidelines), direct overspray scenarios in shallow waterbodies (1–10 m depth) yield PECs of 1–40 µg/L (see table below). These estimates align with concentrations used in ecotoxicological studies to simulate realistic contamination events.

waterbody depth (m)	waterbody volume per m2 in L	g fipronil per m2	g fipronil per m2	ug/m2	ug/m2	ug/L	ug/L
		100 g/ha	500 g/ha	100 g/ha	500 g/ha	100 g/ha	500 g/ha
1	1000	0.01	0.04	10000	40000	10	40
2	2000	0.01	0.04	10000	40000	5	20
5	5000	0.01	0.04	10000	40000	2	8
10	10000	0.01	0.04	10000	40000	1	4

b) Field Experiments Simulating Realistic Conditions:

In sugarcane field experiments, fipronil concentrations in adjacent ponds (via runoff) reached 0–0.4 µg/L (Schiesari et al. 2023; Freitas et al. 2024). A mesocosm study mimicking sugarcane field contamination (500 g/ha application) reported initial concentrations of 43 µg/L, declining to 3 µg/L after 14 days and 0.1 µg/L after 75 days (Cotta et al. 2023).

c) Field Measurements in Contaminated Waterbodies:

Environmental monitoring has detected fipronil at 0.05–26.2 µg/L downstream of rice fields

(Marchesan et al. 2010) and <12.6 μg/L in residential areas (Gan et al. 2012), confirming its presence in surface waters at concentrations overlapping with our experimental dose.

d) Rationale for 2 μg/L in This Study:

Our concentration of 2 μg/L falls within the range of measured and predicted field concentrations. Importantly, our pilot experiment confirmed that this level induces detectable changes in zooplankton abundances (e.g., shifts in dominance, reduced richness) without causing widespread extinctions, a critical balance for studying metacommunity recovery dynamics.

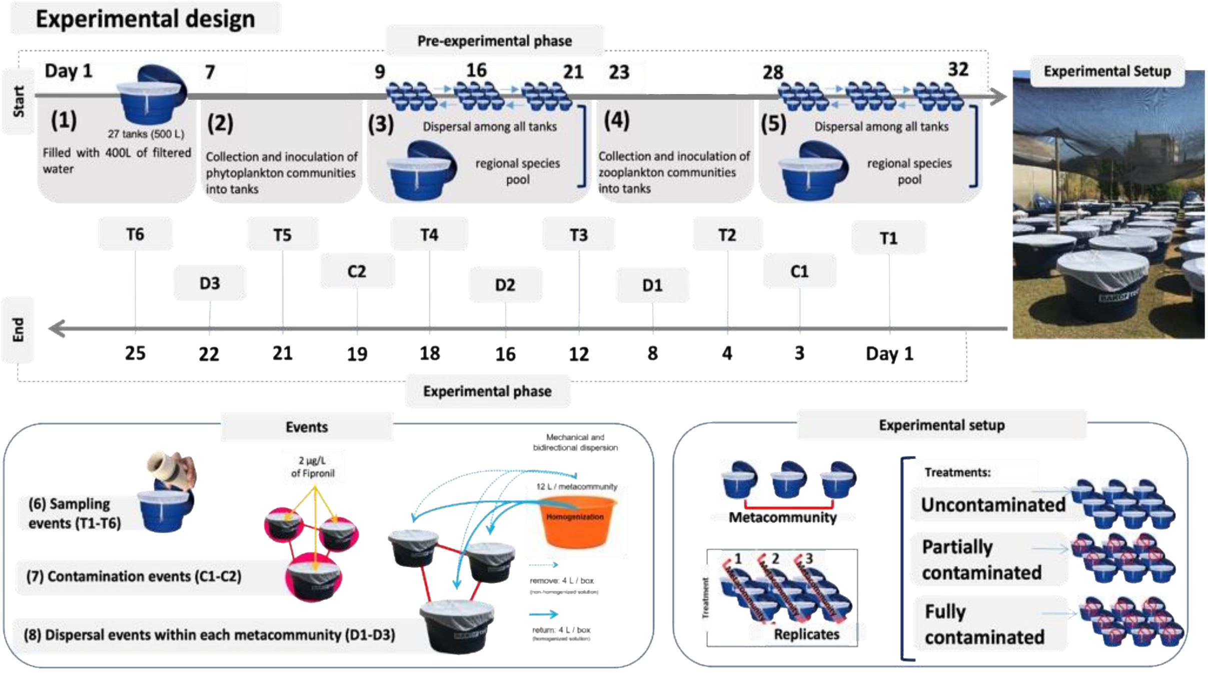

Figure S1. Overview of the set-up and phases of the experiment to understand the reorganization of communities and metacommunities. We simulated contamination gradients in metacommunities using the pesticide fipronil. The pre-experimental and experimental phases included 8 stages: (1) Setting up the mesocosms with polypropylene tanks filled with 400 litres of water and organized into metacommunities, formed by sets of three tanks; one tank represented a community and three tanks represented a metacommunity. (2) Inoculation of the phytoplankton and zooplankton communities. (3) Homogenization of the community composition, via multidirectional dispersal among all tanks, to ensure that all the tanks shared maximum similarity in taxonomic composition. (4) Temporal sampling of the communities divided into six events throughout the experiment (T1-T6), done by filtering 30 L of water in each of the boxes with a 0.20 µm mesh. (5) Contamination, applied at the nominal concentrations of 2 µg/L of fipronil in each tank, according to specific treatments. (6) Dispersal among the three communities of each simulated metacommunity by removing 4 L of water from each tank, which were homogenized and returned to the tanks. This procedure was repeated for all the metacommunities. Image credits: all photographs taken and owned by Victor S. Saito.

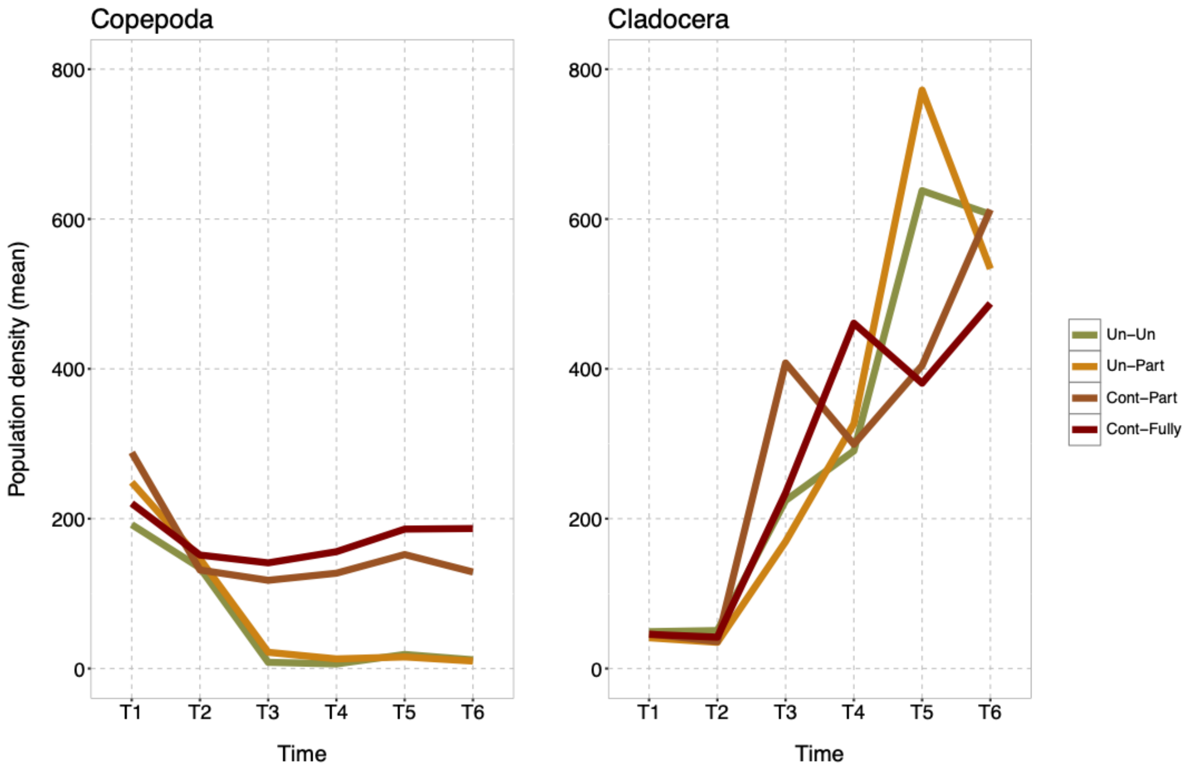

Figure S2. Density of Cladocera and Copepoda in the experimental mesocosms. Lines represent mean density across water tanks as per the following combination of local and regional contamination treatments: local uncontaminated in uncontaminated metacommunities (Un-Un), local uncontaminated in partially contaminated metacommunities (Un-Part), local contaminated in partially contaminated metacommunities (Cont-Part), local contaminated in fully contaminated metacommunities (Cont-Fully).

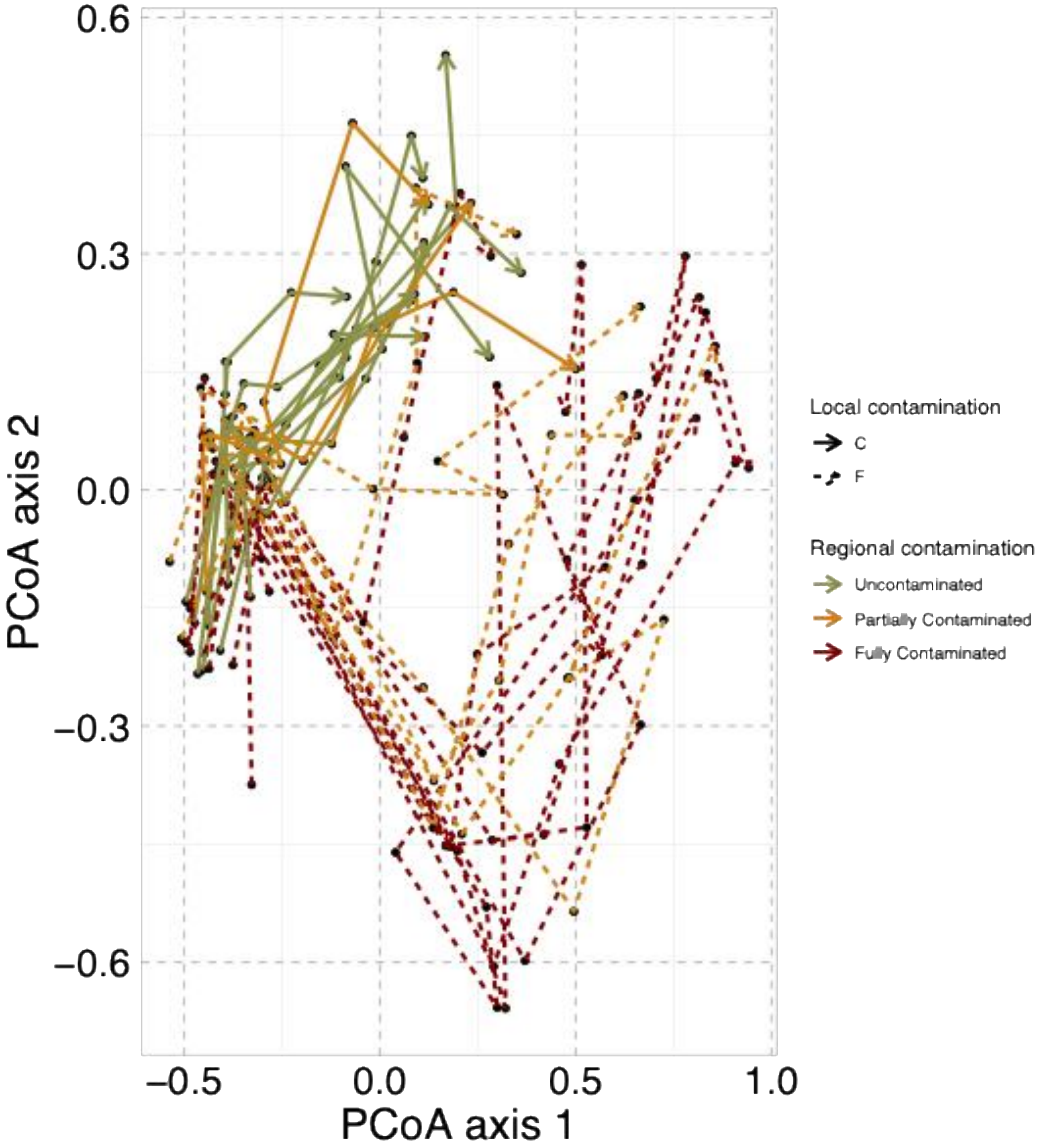

Figure S3. Principal coordinate analysis plot depicting the trajectories of communities in experimental mesocosms using Bray-Curtis dissimilarity. Each treatment was composed of three replicated metacommunities, each one with three communities connected by dispersal. There were two contaminated and one uncontaminated local community in partially contaminated metacommunities (dashed and solid lines, respectively). Metacommunities and whole treatment trajectories were presented using the summed abundance in local communities. Arrows represent the direction of community change following six sampling events along the experiment.

Table S1. Comparison of trajectory lengths at the community level. Summary statistics of specific pairwise comparisons made a posteriori (contrasts) of the generalized linear models (GLM) with Gaussian distributions. P-values were estimated based on 23 degrees of freedom. The letters C (control) and F (Fipronil) indicate the local treatment within the metacommunity treatments.

**Contrast**	**Estimate**	**SE**	**T-ratio**	**P-value**
C Partially contaminated - F Partially contaminated	-0.659	0.202	-3.268	**0.017**
C Partially contaminated - C Uncontaminated	0.015	0.190	0.081	1.000
C Partially contaminated - F fully contaminated	-0.918	0.190	-4.829	**0.000**
F Partially contaminated - C Uncontaminated	0.675	0.150	4.488	**0.001**
F Partially contaminated - F fully contaminated	-0.259	0.150	-1.724	0.335
C Uncontaminated - F fully contaminated	-0.934	0.134	-6.944	**0.000**

Table S2. Comparison of trajectory directionality. Summary statistics of specific pairwise comparisons made a posteriori (contrasts) of the generalized linear models (GLM) with Gaussian distributions. P-values were estimated based on 23 degrees of freedom. The letters C (control) and F (Fipronil) indicate the local treatment within the metacommunity treatments.

**Contrast**	**Estimate**	**SE**	**T-ratio**	**P-value**
C Partially contaminated - F Partially contaminated	-0.005	0.038	-0.124	0.999
C Partially contaminated - C Uncontaminated	-0.014	0.036	-0.394	0.979
C Partially contaminated - F fully contaminated	0.059	0.036	1.645	0.374
F Partially contaminated - C Uncontaminated	-0.009	0.029	-0.332	0.987
F Partially contaminated - F fully contaminated	0.064	0.029	2.248	0.140
C Uncontaminated - F fully contaminated	0.074	0.026	2.884	**0.039**

Table S3. Changes in species richness. Summary statistics of specific pairwise comparisons made a posteriori (contrasts) of the generalized linear models (GLM) with Gaussian distributions. When the interaction between time and contamination was associated with a $p < 0.05$, we compared the treatments with each other at the initial (T1) and final (T6) sampling events. When the interaction between time and contamination was associated with a $p > 0.05$ and contamination was associated with a $p < 0.05$, we compared only the contamination treatments with each other, regardless of time. When only time was associated with $p < 0.05$, we did not make comparisons between times. P-values were estimated based on 6 degrees of freedom. SE: standard error.

**Contrast**	**Estimate**	**SE**	**T-ratio**	**P-value**
Copepoda				
Local				
Partially contaminated - Uncontaminated	-0.2366	0.123	-1.931	0.1017
Partially contaminated - fully contaminated	0.0817	0.123	0.667	0.5295
Uncontaminated - fully contaminated	0.3184	0.123	2.598	**0.0408**
Regional				
Partially contaminated - Uncontaminated	0.0324	0.107	0.304	0.7717
Partially contaminated - fully contaminated	0.3036	0.107	2.844	**0.0294**
Uncontaminated - fully contaminated	0.2712	0.107	2.541	**0.044**

Table S4. Pairwise comparison in gamma diversity. Summary statistics of specific pairwise comparisons made a posteriori (contrasts) of the generalized linear models (GLM) with Gaussian distributions. When the interaction between time and contamination was associated with a $p < 0.05$, we compared the treatments with each other at the initial (T1) and final (T6) sampling events. When the interaction between time and contamination was associated with a $p > 0.05$ and contamination was associated with a $p < 0.05$, we compared only the contamination treatments with each other, regardless of time. When only time was associated with $p < 0.05$, we did not make comparisons between times. SE: standard error; Df: degrees of freedom.

**Comparison**	**Estimate**	**SE**	**Df**	**T-ratio**	**P-value**
All					
T1_Uncontaminated – T1_Totally contaminated	0.222	0.543	36	0.409	0.9919
T6_Uncontaminated – T6_Totally contaminated	2.111	0.543	36	3.888	**0.0024**
T1_Uncontaminated – T1_Partially contaminated	-0.202	0.543	36	-0.371	0.9944
T6_Uncontaminated – T6_Partially contaminated	2.232	0.543	36	4.111	**0.0012**
T1_Totally contaminated – T1_Partially contaminated	-0.424	0.543	36	-0.78	0.917
T6_Totally contaminated – T6_Partially contaminated	0.121	0.543	36	0.223	0.9992
Cladocera					
T1_Uncontaminated – T1_Totally contaminated	0.303	0.227	36	1.335	0.6155
T6_Uncontaminated – T6_Totally contaminated	0.501	0.227	36	2.204	0.1586
T1_Uncontaminated – T1_Partially contaminated	0.163	0.227	36	0.716	0.938
T6_Uncontaminated – T6_Partially contaminated	0.803	0.227	36	3.533	**0.0065**
T1_Totally contaminated – T1_Partially contaminated	-0.141	0.227	36	-0.619	0.9626
T6_Totally contaminated – T6_Partially contaminated	0.302	0.227	36	1.329	0.6193

Copepoda					
Uncontaminated – Partially contaminated	0.035	0.194	46	0.18	0.9823
Uncontaminated – Totally contaminated	0.546	0.194	46	2.811	**0.0194**
Partially contaminated – Totally contaminated	0.511	0.194	46	2.631	**0.0304**

References

Cotta, C. P., T. J. S. Pinto, M. P. C. Yoshii, L. C. M. Silva, A. P. Ogura, G. V. M. Gabriel, L. C. Schiesari, J. B. Carmo, C. C. Montagner, E. L. G. Espíndola, and R. A. Moreira. 2023. Exposure to fipronil, 2,4-D and vinasse influences macroinvertebrate assemblage structure: An experimental mesocosm approach. Science of The Total Environment 888:164259.

Gan, J., S. Bondarenko, L. Oki, D. Haver, and J. X. Li. 2012. Occurrence of fipronil and its biologically active derivatives in urban residential runoff. Environmental Science & Technology 46:1489–1495.

Freitas, I. B. F., P. J. D. Neto, L. F. de P. Lopes, M. P. C. Yoshii, L. Girotto, G. V. de M. Gabriel, L. R. Sorigotto, J. B. do Carmo, C. C. Montagner, L. C. Schiesari, L. A. Martinelli, and E. L. G. Espíndola. 2023. Soil management effects of extensive pastures, intensive pastures and sugarcane crops on the availability of metals and nutrients in freshwater: A realistic mesocosm approach. Agriculture, Ecosystems & Environment 350:108473.

Schiesari, L., V. Saito, J. Ferreira, F. Ls, G. Aj, L. Jp, J. C. Oliveira, R. M. Pelinson, B. B. Querido, J. Carmo, and E. L. Espíndola. 2023. Community reorganization stabilizes freshwater ecosystems in intensively managed agricultural fields. The Journal of Applied Ecology 60:1327–1339.
